# Mitigating genetic instability caused by the excision activity of the *phi*C31 integrase in *Streptomyces*

**DOI:** 10.1128/aem.01812-24

**Published:** 2024-12-20

**Authors:** Yadan Duan, Zhangliang Liu, Xiaofang Huang, Lu Xu, Xianxue Wang, Hao Liu, Zhoujie Xie

**Affiliations:** 1 MOE Key Laboratory of Industrial Fermentation Microbiology, College of Biotechnology, Tianjin University of Science and Technology 534786 , Tianjin, China; 2 Tianjin Engineering Research Center of Microbial Metabolism and Fermentation Process Control, Tianjin University of Science and Technology 66345 , Tianjin, China; Indiana University Bloomington, Bloomington, Indiana, USA

**Keywords:** integrase, directionality, genetic stability, genome engineering, *Streptomyces*

## Abstract

Over the past three decades, the integrase (Int) from *Streptomyces* phage *phi*C31 has become a valuable genome engineering tool across various species. *phi*C31 Int was thought to mediate unidirectional site-specific integration (*attP* × *attB* to *attL* and *attR*) in the absence of the phage-encoded recombination directionality factor (RDF). However, we have shown in this study that Int can also catalyze reverse excision (*attL* × *attR* to *attP* and *attB*) at low frequencies in *Streptomyces lividans* and *Escherichia coli*, causing genetic instability in engineered strains. To address this issue, we developed a two-plasmid co-conjugation (TPC) system. This system consists of an *attP*-containing integration vector and an Int expression suicide plasmid, both carrying *oriT* to facilitate efficient conjugation transfer from *E. coli* to *Streptomyces*. Using the TPC system, genetically stable integrants free of Int can be generated quickly and easily. The indigoidine-producing strains generated by the TPC system exhibited higher genetic stability and production efficiency compared to the indigoidine-producing strain generated by the conventional integration system, further demonstrating the utility of the TPC system in the field of biotechnology. We anticipate that the strategies presented here will be widely adopted for stable genetic engineering of industrial microbes using phage integrase-based integration systems.

**IMPORTANCE:**

Large serine recombinases (LSRs), including the bacteriophage *phi*C31 integrase, were previously thought to allow only unidirectional site-specific integration (*attP* × *attB* to *attL* and attR). Our study is the first to show that the *phi*C31 integrase can also catalyze a low-efficiency reverse excision reaction in *Streptomyces* and *E. coli* without the involvement of the phage-encoded recombination directionality factor (RDF). The genetic instability caused by the low *in vivo* excisionase activity of the *phi*C31 integrase is a major challenge for biotechnological applications. Our study addresses this issue by developing a two-plasmid co-conjugation (TPC) system that facilitates the construction of Int-deficient genomic engineering strains. The Int-deficient integrants produced by this TPC system exhibit strong genetic stability for introduced genes and maintain stable production traits even in the absence of selection pressure, making them highly valuable for industrial applications.

## INTRODUCTION

Bacteria of the genus *Streptomyces* are excellent sources of many bioactive compounds with antibacterial, antifungal, herbicidal, immunosuppressive, and antitumor properties (1, 2). Approximately 70% of naturally occurring bioactive compounds with pharmacological and agricultural applications are estimated to originate from Actinomycetes, primarily from the *Streptomyces* genus (3, 4). In addition, as one of the major bacterial groups commonly found in soil, *Streptomyces* with naturally versatile protein secretion capabilities, including *Streptomyces lividans*, are attractive hosts for recombinant protein production (5, 6). Because of their importance in biotechnology and medicine, efficient and stable genome modification techniques for *Streptomyces* are desired to activate their production or to manipulate their biosynthesis for increased production or improved properties.

Numerous vector systems are available for the introduction of heterologous genes or gene clusters into *Streptomyces* hosts, including replicative vectors containing the replication origin of *Streptomyces* and integrating vectors designed to insert themselves into specific sites within the genome through recombination mechanisms (7–9). Replicative vectors are of limited use for the efficient introduction and stabilization of large DNA fragments due to plasmid instability. They require continuous antibiotic selection for stable maintenance in *Streptomyces*, complicating their use in biotechnology applications (10). To achieve stable genome editing, suicidal nonreplicating vectors that integrate into the *Streptomyces* genome by homologous recombination (HR) have been developed (11, 12). However, the main disadvantage of HR-based genome editing systems is their low integration efficiency, which depends on the size of the homology arms. Unlike HR-based integration, site-specific recombination-based integration is much more effective, and even large DNA fragments like the phage genome, consisting of up to hundreds of kilobases, can be effectively integrated into the host genome through site-specific recombination (13). Therefore, integrating vectors that site-specifically integrate into the genome using bacteriophage integrases provide an effective means for integrating DNA fragments at specific genomic locations. Integrases (Int) from *Streptomyces* temperate phages phiC31, phiBT1, R4, and TG1 have been used to develop versatile vectors capable of integration into different *attB* sites in *Streptomyces* (13). These include the widely used *phi*C31 Int-based integration vector pSET152 and the phiBT1 Int-based vector pIJ10500 (14, 15). Among these, integration plasmids derived from bacteriophage *phi*C31 are the most commonly used in *Streptomyces* (16).

The *phi*C31 Int mediates site-specific DNA recombination between the bacterial attachment site (*attB*) and the phage attachment site (*attP*) without requiring other accessory proteins, auxiliary DNA sequences, or specific DNA topologies, generating *attL* and *attR* sites that flank the integrated prophage (17). The reaction catalyzed by *phi*C31 is highly directional: excision recombination (between *attL* and *attR*) in the absence of accessory factors is generally not observed (18). The gp3 protein, a *phi*C31 early protein that acts as a recombination directionality factor (RDF), could bind to Int in solution and convert the activity of the *phi*C31 Int into an excisionase (catalyzing *attL* × *attR* recombination, leading to *attP* and *attB* products) (19, 20). Indeed, the demonstrated unidirectional characteristic for *phi*C31 Int is particularly well-suited for the stable integration of DNA, compared to bidirectional recombinases such as Cre and FLP (21). Therefore, another major advantage of these vectors derived from *phi*C31 integrase was believed to be their stability. The unique properties of *phi*C31 have enabled its development into various genome engineering tools across diverse cellular systems, beyond *Streptomyces*, including prokaryotes, eukaryotes, and archaea (22–24).

Here, by developing a sensitive excision detection assay based on a recombinant selection marker, we demonstrated that integrants generated by the conventional *phi*C31 Int-based integration system are genetically unstable, with integration vectors being excised from the genome at a low but significant frequency because of the *in vivo* excision reaction caused by Int, leading to reduction in biotechnological properties of the engineered strains. To address these issues, we developed a two-plasmid co-conjugation (TPC) system to create integrase-free integrants. The system consists of a cargo integration vector containing an *attP* site and a suicide helper plasmid that transiently expresses Int. Using the TPC system, integration of foreign DNA at the *attB* site of the recipient strain, without cointegration of the *int* gene, can be easily achieved, and the excision reaction in the resulting integrants is undetectable at the limit of our assay. Furthermore, indigoidine-producing strains, which produce a nonribosomal peptide blue pigment, were created by inserting the indigoidine biosynthetic gene cassette into the genome of *S. lividans* using both the TPC system and the regular pSET152 integration system, respectively. The strains created by the TPC system showed stronger genetic stability and higher production titers compared to those created by the conventional system, demonstrating the utility of the TPC system in the genome engineering of biotechnologically important *Streptomyces*.

## RESULTS

### Recombinant marker design for low-frequency excision detection

The plasmid pSET152, a *phi*C31 Int/attP-based integrating vector widely used in the genetic engineering of *Streptomyces*, can integrate at the *attB* locus within the chromosome of *Streptomyces* host strains (10, 14). During our cultivation of the pSET152 integration strain (*S. lividans* S4098) without antibiotic pressure, we consistently detected a weak DNA band corresponding to the wild-type *attB* locus by PCR from the genomic DNA of the cell culture of *S. lividans* S4098 (Fig. S1). This observation led us to speculate that plasmid excision might occur at a low frequency during cultivation. To detect these putative low-frequency excision events, we designed an assay for the positive selection of excision events based on a recombinant hygromycin resistance gene (*hygB*).

**Fig 1:**
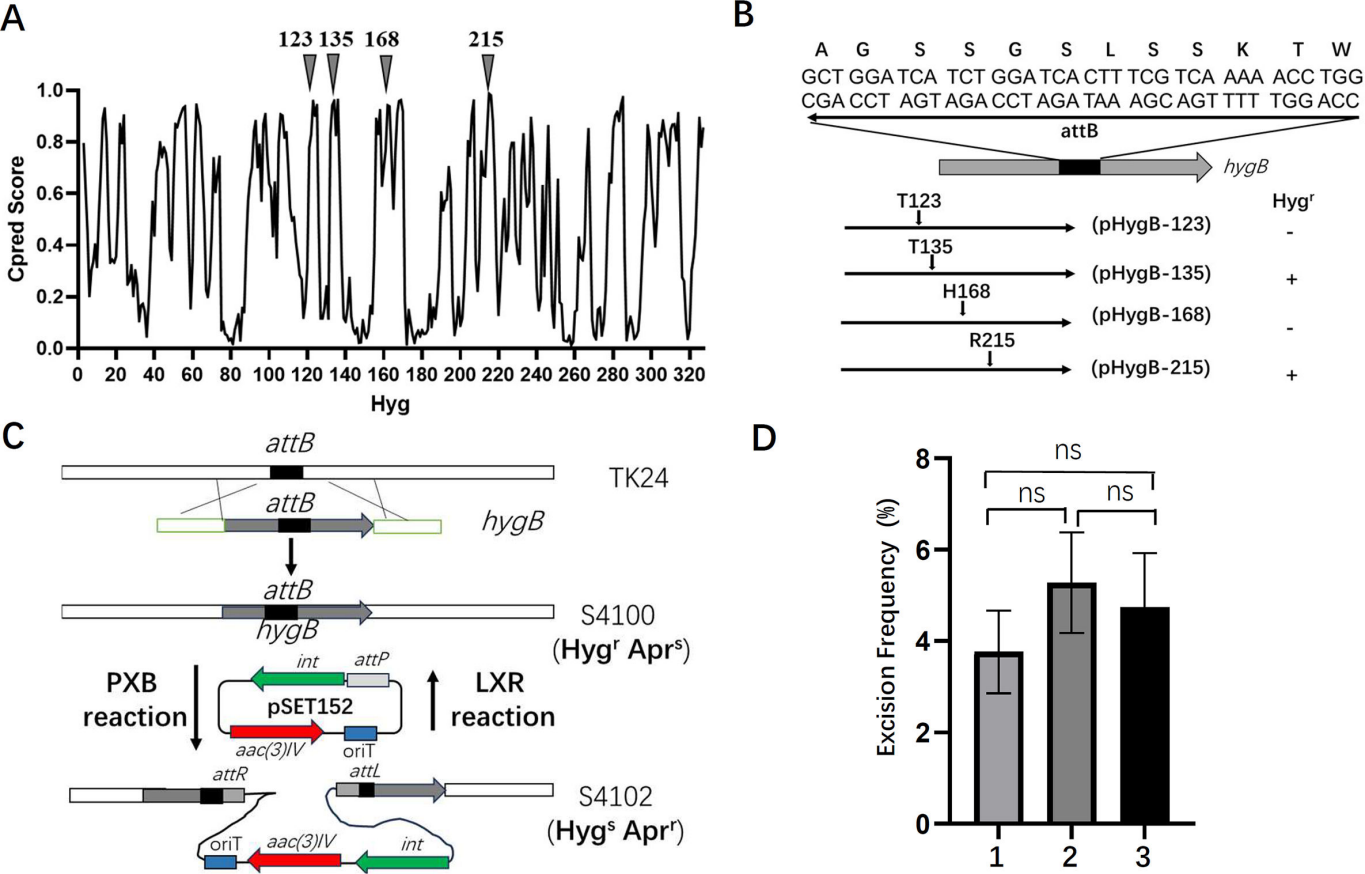
Detection of low-frequency excisions in the pSET152 integration strains. (**A**) Predicted circular permutation (CP) probabilities (CPred scores for Hyg) plotted against residue number. The locations of the predicted Hyg CP sites selected in this study are indicated by closed gray triangles. (**B**) The design of recombinant hygromycin resistance genes containing *attB*. Changes in the *hyg* sequence are shown at the junctions where *attB* is joined to the split sites. The recombinant HygB is thus 12 amino acids longer than the native Hyg. (**C**) Illustration of the *in vivo* excision detection procedure using the recombinant hygromycin resistance gene (*hygB*) as a selection marker. The strain S4100 was generated by replacing the native *attB* of *S. lividans* TK24 with *hygB*. When S4100 was used as the host strain for pSET152 integration (P × B recombination), the resulting integrants (S4102) exhibited a hygromycin-sensitive and apramycin-resistant phenotype (Hyg^s^Apr^r^). Excision of integrated pSET152 in *S. lividans* S4102 (L × R recombination) leads to regeneration of *hygB*, which restores the hygromycin-resistant and apramycin-sensitive phenotype of the host strain (Hyg^r^Apr^s^). (**D**) Excision efficiency as reflected by the frequency of hygromycin resistance recurrence. Verified *S. lividans* S4102 was inoculated into the mannitol soya (MS) plate without addition of antibiotics for sporulation. The number of hygromycin-resistant spores was counted as a percentage of the total number of spores. The results of three independent experiments are shown. ns, no statistically significant difference.

The recombinant hygromycin resistance gene (*hygB*) was engineered from the wild-type hygromycin resistance gene (*hyg*) of *Streptomyces hygroscopicus* (25) by inserting an *attB* site into the *hyg* gene without disrupting the open-reading frame. Since *hygB* is expected to retain the same function as the original *hyg* gene, identifying suitable split sites that can accommodate the insertion of the protein linker encoded by *attB* without compromising the gene’s function was essential for the successful design of *hygB*. Previous research has demonstrated that valid circular permutation (CP) sites in proteins can serve as potential split sites for creating split proteins (26). Following a similar approach, we aimed to identify an internal permissive split site within Hyg. The solution structure of Hyg was determined (27), allowing us to estimate the CP probability as a function of the residue number using CPred (28), an online CP prediction tool. Based on these predictions, four CP sites with the highest scores (corresponding to residues 123, 135, 168, and 215) were selected as candidate split sites (Fig. 1A). Accordingly, four recombinant resistance genes were constructed by inserting the *attB* site after each of these split sites. Hygromycin resistance screening revealed that two constructs—the recombinant *hyg* gene with *attB* inserted after the 135^th^ codon and the recombinant *hyg* gene with *attB* inserted after the 215^th^ codon—retained hygromycin resistance function (Fig. 1B), indicating that Hyg activity was restored in both cases. The recombinant *hyg* gene with *attB* inserted after the 215^th^ codon was designated as *hygB* and used in subsequent studies.

Starting with *S. lividans* TK24 as the parent strain, we generated *S. lividans* S4100 by replacing the native *attB* site with the recombinant *hygB* gene (Fig. S2). As a result, there is still one *attB* site in the genome of *S. lividans* S4100, located within the *hygB* gene. Integration of pSET152 (which contains apramycin resistance gene as a selection marker) into the *attB* site of strain S4100 results in the disruption of *hygB*, causing the resulting integrant, S4102 (Fig. S3), to exhibit apramycin resistance and hygromycin sensitivity. When the integrated vector is excised from the genome of S4102, the hygromycin resistance phenotype will be restored (Fig. 1C). This allows us to quantitatively monitor the frequency of vector excision by measuring the reoccurrence of hygromycin resistance. Three colonies of *S. lividans* S4102 were selected to assess the reoccurrence frequency of hygromycin resistance during cultivation on the MS plate without antibiotics, which was found to be approximately 4.5% (Fig. 1D). Further analysis of the emerging hygromycin-resistant strains through PCR and sequencing confirmed the reappearance of *hygB* (data not shown), indicating the occurrence of vector excision.

### *In vivo* excision of integrated pSET152 is dependent on Int

**Fig 2:**
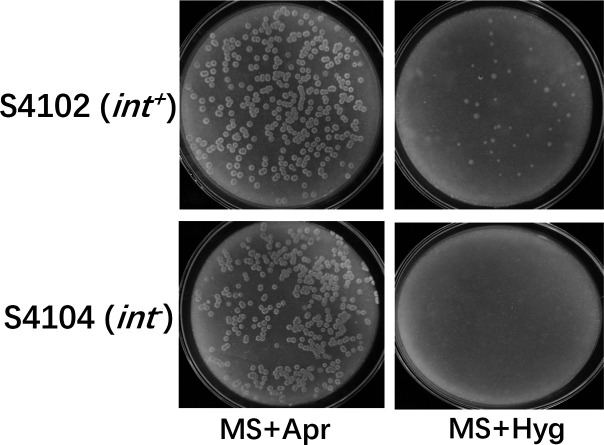
Excision of integrated pSET152 is dependent on the presence of *int*. Excision detection in pSET152 integration strain *S. lividans* S4102 and *int* deletion strain *S. lividans* S4104. Both strains S4102 and S4104 were grown on MS agar without the addition of antibiotics for sporulation. For each strain, the collected spores were plated directly (without further passage) on MS agar with apramycin (MS +Apr) and MS agar with hygromycin (MS +Hyg) in parallel. Hygromycin-resistant colonies were obtained from *S. lividans* S4102, indicating the presence of *in vivo* excision, whereas hygromycin-resistant colonies were never obtained from *S. lividans* S4104, indicating the absence of *in vivo* excision

To determine whether *phi*C31 Int was responsible for the excision process in strain S4102, the gene *int* introduced by the integration of pSET152 was deleted from strain S4102 as described in Materials and Methods to obtain strain S4104 (Fig. S4). The excision frequency of the resulting strain S4104 was monitored by the reoccurrence frequency of hygromycin resistance during cultivation on the MS plate without antibiotics. As shown in Fig. 2, no cells with the hygromycin-resistant phenotype were detected, indicating that plasmid excision did not occur at all in strain S4104. In contrast, a significant number of hygromycin-resistant strains were observed for the parent strain S4102 on the hygromycin plate (Fig. 2). This result indicates that the excision of integrated pSET152 in strain S4102 is dependent on *int*. Correspondingly, RT-PCR experiments showed that *int* transcripts could not be detected in strain S4104, while the expression of *int* was clearly detectable in strain S4102 (Fig. S5). Thus, even though the *attP* site is located within the *int* promoter region in plasmid pSET152, the vector integration does not abolish the expression of *int* in strain S4102.

### *In vivo* reverse recombination catalyzed by *phi*C31 Int in *E. coli*

Previous studies have suggested that Int alone, without other phage proteins, cannot recombine the *attL* and *attR* sites *in vivo* in *E. coli*. These low-frequency recombination events might have been overlooked because the excision assay previously used was based on a blue–white color change using a reporter plasmid containing *attL* and *attR* flanking a *lacZ*α gene (19), which is not sensitive enough. To detect these low-frequency recombination events, we designed a novel excision assay for positive selection of the excision reaction in *E. coli* based on the regeneration of the recombinant hygromycin resistance gene-*hygB*.

**Fig 3:**
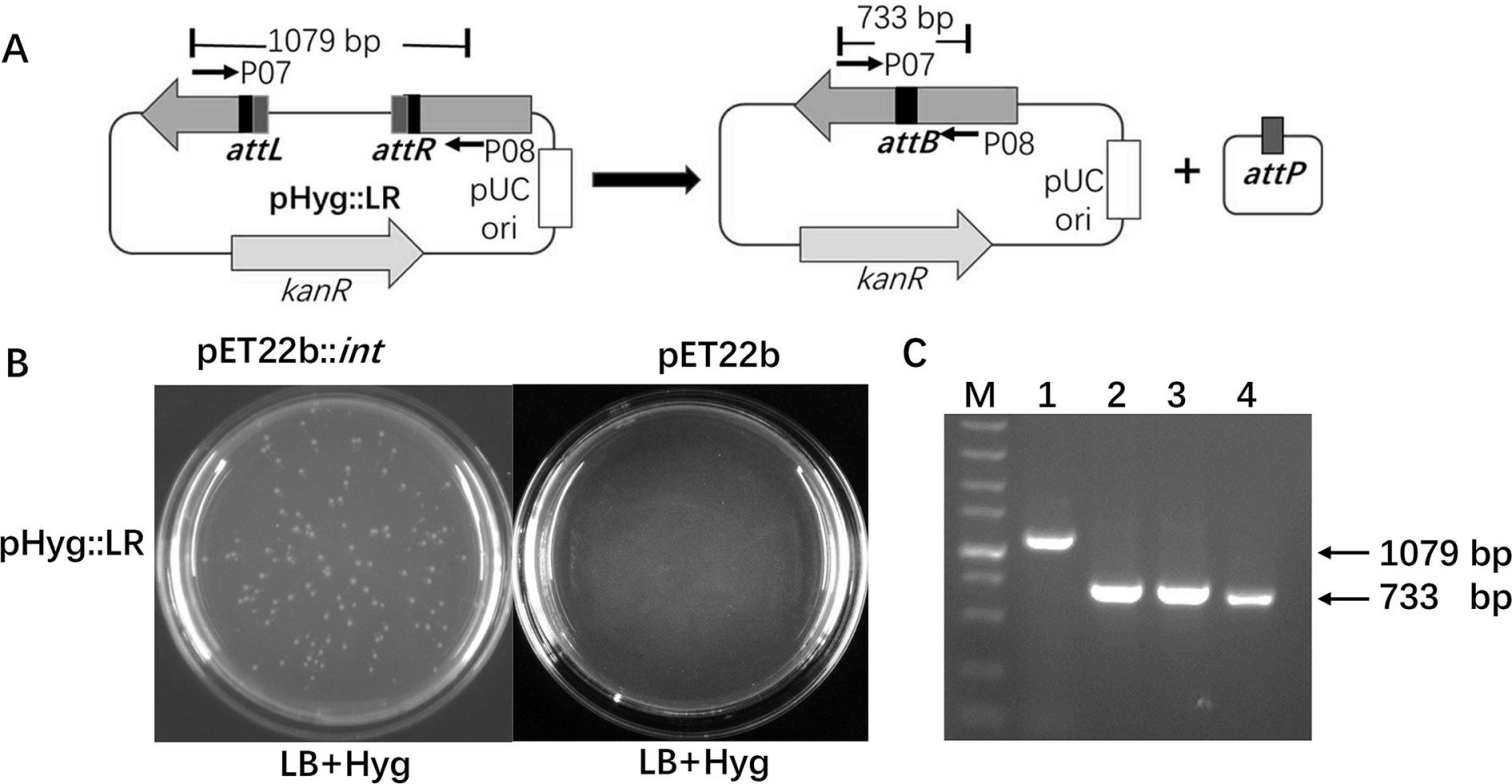
The excision recombination activity of *phi*C31 Int on the *attR* × *attL* substrate in *E. coli*. (**A**) Illustration of the strategy using the reporter plasmid pHyg::LR to detect *in vivo attL* × *attR* recombination. The reporter plasmid pHyg::LR harbors an inactivated *hyg* gene with the insertion of a DNA fragment flanked by *attL* and *attR* after the 215^th^ codon of *hyg*. The *attL* ×*attR* recombination in pHyg::LR results in the formation of functional *hygB*, conferring hygromycin resistance to the cells. (**B**) *E. coli* cells containing both the reporter plasmid pHyg::LR and the Int expression vector pET22b::*int* were grown for 20 hours in Luria–Bertani (LB) medium containing ampicillin, kanamycin, and IPTG. After that, the culture was spread on LB agar containing hygromycin. Hygromycin-resistant colonies were obtained, indicating that excision had occurred. In contrast, cells containing pHyg::LR and the empty vector pET22b, cultivated under the same conditions, did not yield hygromycin-resistant colonies, indicating no *attL* ×*attR* recombination. (**C**) PCR analysis of pHyg::LR and recombinant plasmids extracted from hygromycin-resistant colonies described above. The primer pair P07/P08, which spans the insertion site of the LR fragment in pHyg::LR (as indicated by arrows in Fig. 3A), was used. Lane M: 1-kb DNA marker. A single band corresponding to the recombinant product *hygB* (733 bp) was produced for the plasmids extracted from three randomly selected hygromycin-resistant colonies (lanes 1, 2, and 3).

A reporter plasmid, pHyg::LR, and an Int-expressing plasmid, pET22b::*int*, were used in the assay. In pHyg::LR, a DNA fragment flanked by *attL* and *attR* was inserted into the hygromycin resistance gene *hyg* after the 215^th^ codon, resulting in an inactivated hygromycin resistance gene *hyg::LR*. The *attL* and *attR* sites in pHyg::LR are arranged in a head-to-tail orientation, so recombination between *attL* and *attR* will result in the excision of the insertion and generation of a functional *hygB* gene, conferring hygromycin resistance (Fig. 3A). pET22b::*int* is a *phi*C31 Int-expressing plasmid using pET22b as a vector. When *E. coli* BL21(DE3) cells harboring both pET22b::*int* and pHyg::LR were cultivated in the presence of kanamycin and ampicillin to maintain the coexistence of both recombinant plasmids, along with IPTG to induce *int* expression, hygromycin-resistant colonies were observed when the culture was plated on Luria–Bertani (LB) agar containing hygromycin (Fig. 3B). As a control, when the same assay was performed with *E. coli* BL21(DE3) containing the empty vector pET22b and the excision reporter plasmid pHyg::LR, no hygromycin-resistant colonies were observed (Fig. 3B). Further plasmid extraction and PCR analysis showed that plasmids containing *hygB* could be recovered from the hygromycin-resistant colonies (Fig. 3C). The regeneration of *hygB* from the nonfunctional *hyg::LR* indicates that recombination between *attL* and *attR* occurred in the cells when Int was co-expressed.

### Purified *phi*C31 Int fails to catalyze *in vitro* reverse recombination

In this study, both integration and excision assays were performed *in vitro* using purified *phi*C31 Int. Consistent with the previous report (17), our results showed that *phi*C31 Int can efficiently catalyze the *in vitro* recombination reaction between *attP* and *attB* (Fig. S6). *In vitro attL* × *attR* recombination was assayed using plasmid pHyg::LR as the substrate. Successful recombination would generate the hygromycin-resistant plasmid pHygB-215. However, the reverse excision reaction between *attL* and *attR* could not be detected by PCR (Fig. S6). Further analysis was performed by introducing the complete reaction mixture into *E. coli* JM109 *via* transformation and selecting for transformants carrying pHygB-215 on hygromycin-containing media. The results showed that no hygromycin-resistant clones were obtained (data not shown). Overall, our excision assay indicates that while *phi*C31 Int is capable of catalyzing recombination between *attL* and *attR in vivo*, it is unable to do so *in vitro* without the presence of additional phage proteins. Based on these results, we speculate that certain host factors may play a role in the *in vivo* excision process catalyzed by *phi*C31 Int.

### A two-plasmid co-conjugation (TPC) system for constructing genetically stable Int-deficient integrants

**Fig 4:**
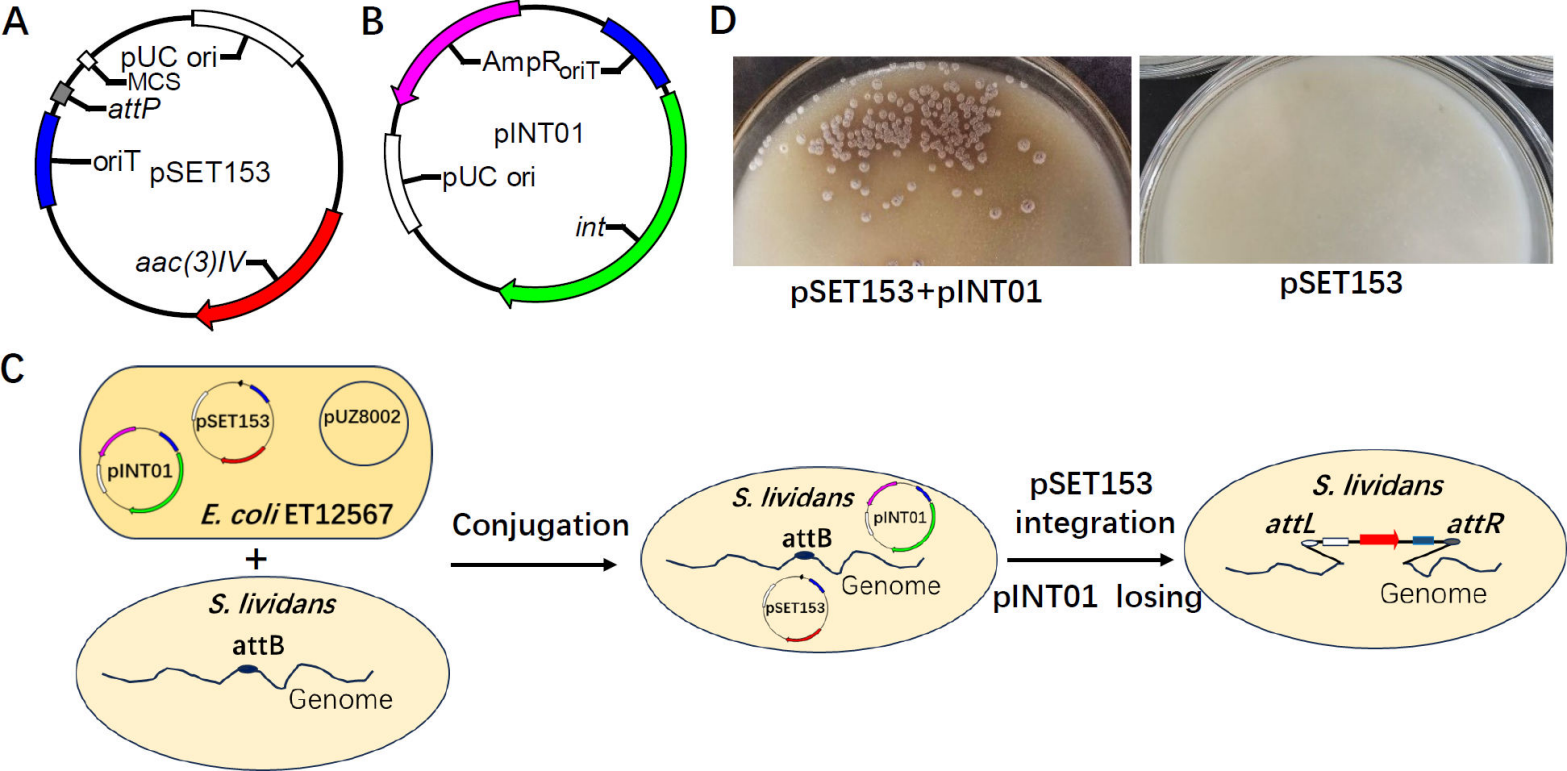
A two-plasmid co-conjugation (TPC) system for the efficient construction of genetically stable Int-deficient integrants. (**A**) Plasmid map of pSET153. Features include the following: *aac(3)IV* (apramycin resistance gene), *oriT* (origin of plasmid transfer), *attP* (attachment site for integration), MCS (multiple cloning site), and pUC ori (origin of replication in *E. coli*). (**B**) Plasmid map of pINT01. Features include the following: *int* (*phi*C31 integrase gene), *ampR* (ampicillin resistance gene), pUC ori (origin of replication in *E. coli*), and *oriT* (origin of plasmid transfer). (**C**) Schematic representation of the Int-deficient integrant construction process through conjugation. Both pSET153 and pINT01 are co-transformed into *E. coli* ET12567(pUZ8002). The donor strain, *E. coli* ET12567(pSET153, pINT01, pUZ8002), can transfer pSET153 and pINT01 to the recipient *Streptomyces* strain via conjugation. Transient expression of *phi*C31 *int* from pINT01, which is lost during subsequent cell divisions due to lack of an origin of replication, allows the integration of the vector pSET153 into the *attB* site of the host strain. (**D**) Using *S. lividans* S4100 as the recipient strain, *E. coli* ET12567(pUZ8002) containing both pSET153 and pINT01 and *E. coli* ET12567(pUZ8002) containing only pSET153 were used as donor strains for conjugation. Only the former efficiently produced ex-conjugates.

The above-mentioned study demonstrates that integrants created using the conventional *phi*C31 att/int integration system are genetically unstable due to the excision of the integrated plasmid. To overcome this problem, we developed a new genome integration system consisting of pSET153 and pINT01. pSET153 is a *Streptomyces* integrative plasmid derived from pSET152 by deletion of the *int* gene (Fig. 4A). pINT01 is a *Streptomyces* suicide plasmid providing integrase *in trans* (Fig. 4B). Both pSET153 and pINT01 contain the origin of transfer (*oriT*) from the plasmid RK2 (IncP) (14, 29). Using *S. lividans* S4100 as the recipient strain, we successfully constructed Int-deficient integrants through the strategy illustrated in Fig. 4C. Briefly, conjugation experiments were carried out between the methylation-deficient *E. coli* donor strain ET12567 (pUZ8002), carrying both pSET153 and pINT01, and the *Streptomyces* recipient strain *S. lividans* S4100 using the co-conjugation method described in Materials and Methods. Apramycin-resistant exconjugants were generated efficiently (the conjugation frequency can reach to 5.0 × 10^-4,^ which is sufficient for normal genetic manipulation) (Fig. 4D). PCR experiments further confirmed that the vector pSET153 was integrated site-specifically into the *attB* site of the recipient strain and pINT01 could not be detected in the resulting strain (designated as S4106) (Fig. S7). In contrast, when the *E. coli* donor strain ET12567 (pUZ8002) carrying only pSET153 was used for the conjugation assay, no apramycin-resistant exconjugants were observed (Fig. 4D). These results indicate that plasmids pSET153 and pINT01 can be co-transferred into the recipient *Streptomyces* through conjugation. The transient expression of *phi*C31 *int* from pINT01, which is lost during the growth of the recipient, provides enough integrases *in trans* to allow for the integration of the vector pSET153.

The genetic stability of the integrated pSET153 in *S. lividans* S4106 was evaluated by determining the reoccurrence frequency of hygromycin resistance, as described in Materials and Methods. As expected, no hygromycin-resistant revertants were observed from the culture of *S. lividans* S4106 (data not shown), indicating the genetic stability of *S. lividans* S4106. In conclusion, the newly developed two-plasmid co-conjugation system offers a versatile tool for the stable integration of heterologous genes in *Streptomyces*.

### The potential of the TPC system for constructing genetically stable and high-yielding strains for biotechnology applications

**Fig 5:**
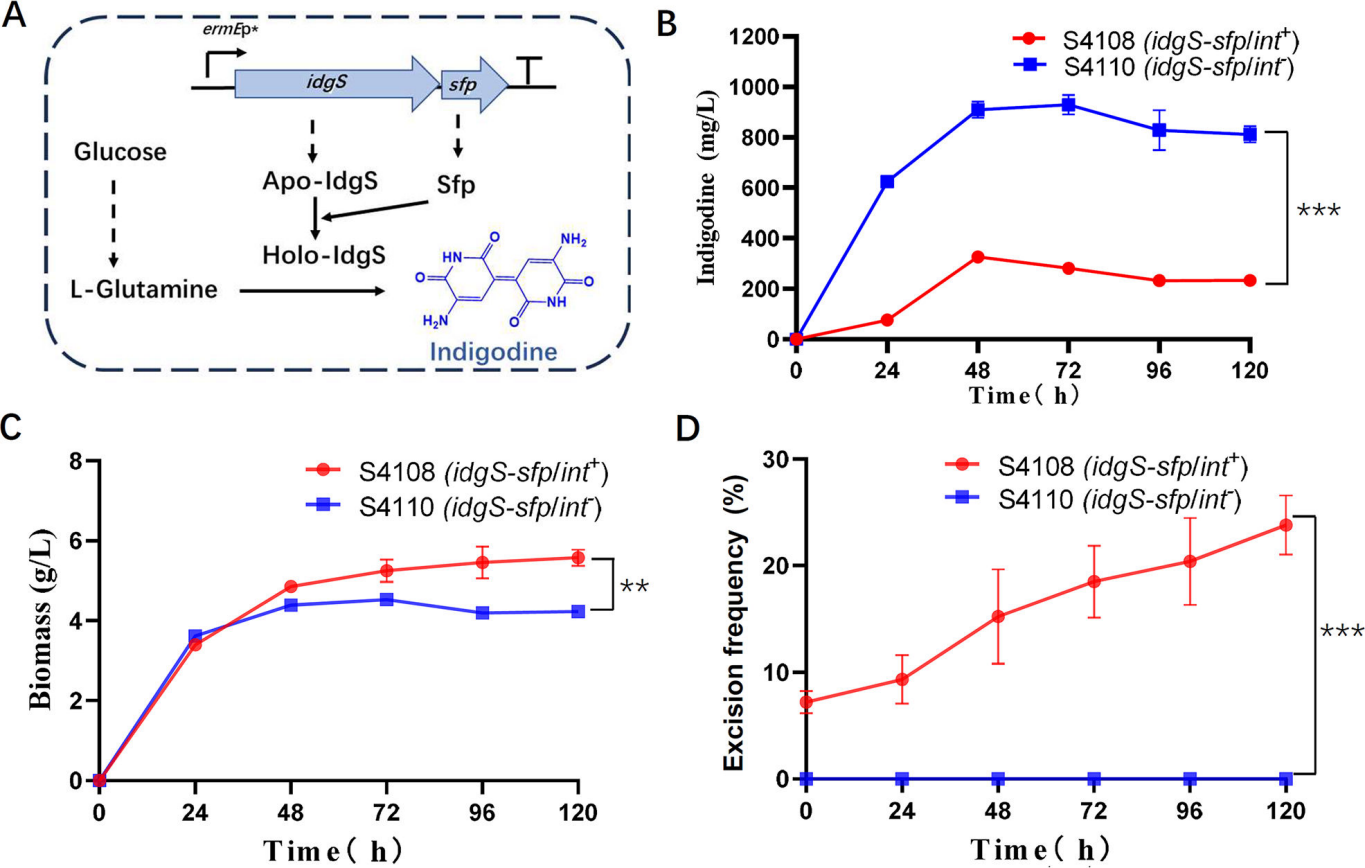
High yield biosynthesis and genetic stability for Int-deficient integrants. (**A**) Overview of indigoidine biosynthesis via the introduction of the *idgS-sfp* operon. (**B**) Production of indigoidine in the Int-proficient genomic integration strain S4108 (*idgS-sfp*/*int*^+^) and the Int-deficient genomic integration strain S4110 (*idgS-sfp*/*int*^-^). (**C**) Growth curves of the Int-proficient genomic integration strain S4108 (*idgS-sfp*/*int*^+^) and the Int-deficient genomic integration strain S4110 (*idgS-sfp*/*int*^-^). (**D**) Excision efficiencies of the integrated plasmid in the Int-proficient genomic integration strain S4108 (*idgS-sfp*/*int*^+^) and the Int-deficient genomic integration strain S4110 (*idgS-sfp*/*int*^-^). Excision efficiencies are reflected by the frequency of hygromycin-resistant reoccurrence. No hygromycin-resistant revertants were observed throughout the fermentation period for strain S4110. **P < 0.001; ***P < 0.0001, as determined by unpaired two-tailed *t-*test.

As a proof of concept to demonstrate the biotechnological potential of the TPC system developed in this study, heterologous indigoidine-producing strains were constructed by inserting indigoidine biosynthetic genes into the genome of *S. lividans* S4100. Indigoidine, a nonribosomal peptide, is a viable blue colorant alternative for colorants in the dye, ink, and pigment industries (30). The biosynthetic genes responsible for indigoidine production, such as the *idgS* gene from *S. lavendulae* CGMCC 4.1386 (31), the *bpsA* gene from *S. lavendulae* ATCC 11924, and *indC* from *S. chromofuscus* ATCC 49982 (32, 33), have been identified previously. Previous studies have shown that the introduction of the *idgS* gene (a nonribosomal peptide synthetase encoding gene from *S. lavendulae*) and the *sfp* gene (a phosphopantetheinyl transferase encoding gene from *Bacillus subtilis*) can establish the indigoidine biosynthetic pathway in various *Streptomyces* hosts (31). In this study, the *idgS-sfp* two-gene operon was introduced into strain S4100 using pSET152 and pSET153 as vectors (Fig. 5A) through the normal intergeneric conjugation assay and the two-plasmid co-conjugation assay, respectively. This resulted in the construction of indigoidine-producing strains S4108 (pSET152::*idgS-sfp*/S4100) and S4110 (pSET153::*idgS-sfp*/S4100) (Fig. S8). Both strains were cultivated in the indigoidine fermentation medium for 5 days without the addition of antibiotics, and indigoidine production was monitored using the method described in Materials and Methods. As shown in Fig. 5B, the engineered strain S4110 exhibited higher indigoidine production than S4108 throughout the fermentation period. The maximum indigoidine production in S4110 was nearly threefold higher than that of *S. lividans* S4108 (Fig. 5B), despite the biomass of the former being less than that of the latter (Fig. 5C).

Strain S4100 was chosen as the host for the insertion of the indigoidine biosynthetic genes, allowing us to monitor excision frequencies based on the reoccurring frequency of hygromycin resistance in the fermentation culture. As shown in Fig. 5D, at the beginning of fermentation, approximately 7.0% of the cells in the culture of strain S4108 exhibit the hygromycin resistance phenotype. This percentage gradually increased to 23% after 5 days of fermentation (Fig. 5D). Meanwhile, no hygromycin-resistant strain was detected for strain S4110 in the culture throughout the 5 days of fermentation, indicating that no plasmid excision occurred in strain S4110 during the fermentation period.

*S. lividans* S4110, created using the TPC system, exhibits both higher productivity and genetic stability compared to *S. lividans* S4108, which was created using the conventional integration system. This suggests that the TPC system has greater potential for constructing genetically stable and high-yielding strains for biotechnological applications.

## DISCUSSION

*phi* C31 Int is known to catalyze unidirectional recombination between the bacteriophage *attP* and chromosomal *attB* sites. This presumed unidirectionality and stability have facilitated the widespread application of the *phi*C31 att/int system in genetic engineering across various organisms, including Streptomycetes, other Actinomycetes, eukaryotes, and archaea (13).

Our study is the first to show that *phi*C31 Int can catalyze *in vivo* excision recombination at low efficiency in both *Streptomyces* and *E. coli. phi*C31 belongs to the large serine recombinases (LSRs). Other well-studied proteins in this group include integrases from the *Streptomyces* phages *phi*BT1 and TG1 (34, 35) and the mycobacterial phages Bxb1 and *phi*Rv1 (36, 37). Recently, over 60 LSRs have been identified from microbial sequencing data and experimentally characterized in human cells (38). Although there is evidence that *phi*BT1 integrase has low *in vitro* excisionase activity on *attL* and *attR* substrates (39), *in vivo* excision activity for these LSRs has never been reported in any of these cases. Previous studies typically used LacZ or fluorescent proteins as reporters in excision assays (19, 38), which are not sensitive enough to detect low-efficiency excision. Therefore, it remains to be tested whether other LSRs exhibit low *in vivo* excisionase activity similar to that of *phi*C31. A positive selection procedure, like the one developed in this study, will be useful for future studies to monitor excision events occurring at low frequencies.

The excision reaction could not be detected in our *in vitro* excision assay using purified integrase, consistent with previous reports. This suggests that some host-encoded factors might be involved in the excision process. Previous studies have shown that *phi*C31 integrases can catalyze *in vitro* cleavage of single-site substrates *attL* and *attR*, with cleavage occurring without synaptic complex formation (40). It is possible that the DNA repair system completes the excision process *in vivo* by rejoining the double-strand breaks caused by the cleavage process. Testing the involvement of the DNA repair system in the excision process would be an interesting direction for future studies.

Our study indicated that *phi*C31 Int is essential for the excision of integrated plasmids. Thus, the loss of the integrated vector can be prevented when the integrase gene is not co-integrated into the genome. We established a new integration system to achieve this by keeping *attP* on the integration vector pSET153 and supplying *int* on a suicide vector pINT01. Both plasmids also contain *oriT*. We found that both plasmids can be transferred together into the same *Streptomyce*s host cell through conjugation from *E. coli*, and exconjugates containing only pSET153 were obtained with high efficiency. Protoplast transformation and conjugation from *E. coli* to *Streptomyces* are the two most commonly used methods for genetic material transformation in *Streptomyces*. While protoplast transformation has been adapted to various *Streptomyces* species, it is laborious and requires specific conditions for the formation and regeneration of protoplasts in different *Streptomyces* strains. In contrast, introducing DNA into *Streptomyces* strains through conjugation from *E. coli* is easy and efficient. The two-plasmid co-conjugation (TPC) system established in this study greatly facilitates the construction process for genome engineering strains with stable genetic integration.

We demonstrated that undesirable genetic heterogeneity exists for the pSET152 integration strains during cultivation because of the excision of the integrated plasmid. Genetic instability of the integrants caused by low-efficiency excision reactions can be problematic in biotechnological applications, especially if there is some selection against integrated DNA. This raises the possibility that integrants will eventually be lost, leading to a loss of production traits. A similar case was observed in our construction of the heterologous indigoidine production strain. For the indigoidine production strain S4108, created using the conventional integration system, more than 20% of cells lost the integrated plasmid after 5 days of fermentation in the absence of antibiotics. These hygromycin-resistant revertants lose the ability to produce indigoidine because of the excision of the integrated plasmid. In contrast, for the indigoidine production strain S4110, created using the TPC system, no plasmid excision was observed throughout the fermentation period under the same conditions. Although this study demonstrated the stable integration of only a two-gene operon in the *S. lividans* host, bacteria of the genus *Streptomyces* are among the most important producers of biologically active secondary metabolites, which are typically synthesized by large biosynthetic gene clusters (BGCs). The strong genetic stability and significantly higher production titers of strain S4110 compared to strain S4108 highlight the great potential of the TPC system established in this study for the efficient and stable genome modification of these BGCs in various *Streptomyces* species, ultimately leading to enhanced production of the corresponding active secondary metabolites.

In conclusion, our study demonstrated that *phi*C31 Int can catalyze the *in vivo* reverse excision reaction at low efficiency, leading to the genetic instability of integrants generated with the conventional *phiC*31 att/int system. We addressed this issue by developing the TPC system, allowing for the rapid and easy construction of genetically stable, Int-deficient integrants. The discovery of *phi*C31 Int’s low *in vivo* excision activity and the strategies used to mitigate the resulting genetic instability will provide valuable insights for developing other serine recombinases as tools in genome engineering.

## MATERIALS AND METHODS

### Bacterial strains, plasmids, primers, and growth conditions

Bacterial strains and plasmids used in this study are listed in Table S1, and primers are listed in Table S2. *Escherichia coli* JM109 was used as a general host strain for routine subcloning. The methylation-deficient *E. coli* ET12567 containing pUZ8002 was used as a host for transferring DNA from *E. coli* to *Streptomyces* by intergeneric conjugation (10). pUZ8002 is a nontransmissible plasmid derived from RK2 that can mobilize *oriT*-containing plasmids in *trans* (10). *E. coli* BL21 (DE3) was used for the expression of the recombinant protein. The *S. lividans* TK24 was the parent strain used in this study. For general purposes, *Streptomyces* strains were cultivated on mannitol soya flour (MS) agar or in yeast extract−malt extract (YEME) liquid medium (10). For indigoidine production, the *Streptomyces* strains were grown in TSB liquid medium (10). The *Streptomyces* strains were maintained at 28°C. General approaches for *E. coli* or *Streptomyces* manipulations followed standard protocols (10). When necessary, the following final antibiotic concentrations were used for the selection of *E. coli* transformants: ampicillin, 100 µg/mL; apramycin, 100 µg/mL; kanamycin, 100 µg/mL; chloramphenicol, 12.5 µg/mL. For selection of *Streptomyces* transformants in MS, the final antibiotic concentrations were as follows: erythromycin, 100 µg/mL; apramycin, 50 µg/mL; hygromycin, 50 µg/mL; nalidixic acid, 25 µg/mL.

### Construction of recombinant plasmids

#### Construction of pUC57::hyg, pHygB-123, pHygB-135, pHygB-168, and pHygB-215

To obtain a functional recombinant hygromycin resistance gene with the insertion of *attB* in its open-reading frame, the plasmid pUC57::*hyg*, containing the wild-type hygromycin resistance gene (*hyg*), was first constructed as follows: A 2.4-kb fragment was amplified using the primer pair pUC57F-BsaI/pUC57R-BsaI with pUC57 as a template. Additionally, a 1,054-bp fragment and a 131-bp fragment were amplified using the primer pairs hygF/hygRDBsaI and hygFDBsaI/hygR, respectively, from the wild-type hygromycin resistance gene in pIJ10500 (15). The three fragments were ligated together using the Golden Gate cloning strategy to obtain the plasmid pUC57::*hyg* (41). The plasmid pHygB-123, carrying a recombinant *hyg* gene with the insertion of *attB* after the 123rd codon, was constructed as follows: A 3.6-kb fragment was amplified using the primer pair 123F-attB/123R-attB with pUC57::*hyg* as the template. The resulting fragment was circularized using the Golden Gate cloning strategy to obtain plasmid pHygB-123. Plasmids pHygB-135, pHygB-168, and pHygB-215, which carry a recombinant *hyg* gene with *attB* inserted after the 135th, 168th, and 215th codons, respectively, were constructed using the same strategy as pHygB-123, with the primers listed in Table S2. When *E. coli* cells harboring the four recombinant plasmids were inoculated on LB plates containing hygromycin, only pHygB-135 and pHygB-215 conferred a hygromycin resistance phenotype to *E. coli*. The functional recombinant hygromycin resistance gene in pHygB-215 was designated as *hygB* and used in the subsequent study.

#### Construction of pHyg::LR

A 3.7-kb fragment was amplified using the primer pair attBHF/attBHR with pHygB-215 as a template. A 148-bp fragment and a 232-bp fragment were amplified using the primer pairs attPHF/inR-HindIII and inF-HindIII/attPHR, respectively, from pSET152. The three fragments were ligated together using the Golden Gate cloning strategy to obtain the plasmid pHyg::LR.

#### Construction of pET22b::int

The coding region of *phi*C31 *int* was amplified by PCR using the primer pair intF-NdeI/intR-XhoI from pSET152 and subsequently digested with *Nde*I and *Xho*I. The resulting fragment was cloned into the corresponding sites of the plasmid pET22b to generate pET22b::*int*.

#### Construction of pSET153

The plasmid pSET152 was digested with *Hin*dIII to release a 4.0-kb fragment, which was then purified from an agarose gel. This fragment was self-ligated to generate pSET153.

#### Construction of pINT01

The *oriT* (origin of transfer) region was generated by PCR using the primer pair oriTF/oriTR from pSET152. The coding region of *int* was amplified with intF-XbaI/intR-SphI, and the *kasO*p* promoter was generated by PCR using the primer pair kasOF/kasOR (42). The *oriT* amplicon was digested with *Eco*RI and *Kpn*I, the *kasO*p* promoter amplicon with *Kpn*I and *Xb*aI, and the *int* amplicon with *Xba*I and *Sph*I. The three fragments were then ligated into the *Eco*RI and *Sph*I sites of the vector pUC19 to generate the plasmid pINT01.

#### Construction of pSET152::idgS-sfp and pSET153::idgS-sfp

A 5.0-kb DNA fragment containing the *idgS-sfp* two-gene operon driven by the *ermE*p* promoter was amplified from pCIMt004 using the primer pair ermF/sfpR (31). The *tfd* transcriptional terminator region was PCR-amplified from pIJ8660 with the primer pair tfd-F/R (43). Overlapping regions between the two amplicons allowed a subsequent overlapping PCR using the primer pair ermF/tfd-R. The resulting amplicon was assembled into the *Eco*RV site of pSET152 using the One-Step Cloning Kit (Vazyme Biotech Co., Ltd., Nanjing, China) to obtain pSET152::*idgS-sfp*. The same final amplicon was assembled into the *Eco*RV site of pSET153 using the One-Step Cloning Kit to obtain pSET153::*idgS-sfp*.

### Two-plasmid co-conjugation assay

When pSET153 and its derivatives were used for conjugation, the helper plasmid pINT01 was required. The optimized protocol consisted of the following steps: The donor *E. coli* ET12567 (pUZ8002) containing both pSET153 and pINT01 was grown in the presence of ampicillin, apramycin, and kanamycin to an optical density (OD_600_) of 0.4–0.6 at 600 nm. The cells were collected and washed twice with LB to remove the antibiotics and then suspended in 1 mL of LB. The recipient *S. lividans* spores were washed twice and suspended in 2× YT broth at a concentration of 10^9^ per mL. The *S. lividans* spores were then heated at 50°C for 10 minutes. Donor and recipient cells were mixed and spread on MS plates and grown for 14–20 hours at 28°C. After incubation, the plates were covered with 1 mL of water containing nalidixic acid and apramycin and incubated at 28°C for 3–5 days until the exconjugants appeared. The frequency of transformation was calculated based on the number of exconjugants on a selective plate divided by the number of recipient cells on a nonselective plate. The average frequency from three independent experiments was calculated.

### Construction of strains

#### Construction of S4098

The *S. lividans* strain S4098 was obtained by transforming pSET152 into *S. lividans* TK24 through normal intergeneric conjugation (10). PCR analysis was then used to confirm the integration of pSET152 in the resulting strains with primer pairs P01/P02 and P03/P04 (Fig. S1). The verified strain was designated as *S. lividans* S4098.

#### Construction of S4100

*S. lividans* S4100, derived from *S. lividans* TK24 by replacing the native *attB* site with *hygB*, was constructed using a blue–white screening-based genetic manipulation system (31). Briefly, a 1.6-kb fragment upstream of the *attB* site was amplified by PCR using the primer pair attBupF/attBupR, and a 1.7-kb fragment downstream of the *attB* site was obtained using the primer pair attBdnF/attBdnR. The *hygB* gene was amplified from pHygB-215 with the primer pair hygF-up/hygR-dn. The three fragments, which had overlapping regions, were combined in a subsequent overlapping PCR using the primer pair attBupF/attBdnR. The resulting amplicon was assembled into the *Bln*I site of pCIMt004 to create pDYD001 using the One-Step Cloning Kit. After introducing pDYD001 into *Streptomyces* TK24 by *E. coli–Streptomyces* conjugation, white exconjugants appeared on MS plates containing hygromycin and nalidixic acid within 3 days. These white colonies were picked and verified as double-crossover mutants by PCR using the primer P05 and P06 (Fig. S2). The double-crossover mutant was designated as *S. lividans* S4100.

#### Construction of *S. lividans* S4102

The *S. lividans* strain S4102 was obtained by transforming pSET152 into *S. lividans* S4100 through normal intergeneric conjugation. PCR analysis was then used to confirm the integration of pSET152 in the resulting strains with primer pairs P03/P07 and P08/P09, as shown in Fig. S3. The verified strain was designated as *S. lividans* S4102.

#### Construction of S4104

*S. lividans* S4104, derived from *S. lividans* S4102 by deleting the *int* gene in the integrated plasmid, was also constructed using the blue–white screening-based strategy as described before (31, 44). Briefly, a 1.5-kb fragment upstream of *int* was amplified by PCR using the primer pair intupF/intupR, and a 1.6-kb fragment downstream of *int* was obtained using the primer pair intdnF/intdnR. These two amplicons, having overlapping regions, were combined in a subsequent overlapping PCR using the primer pair intupF/intdnR. The resulting amplicon was assembled into the *Bln*I site of pCIMt004 to create pDYD002 using the One-Step Cloning Kit. After introducing pDYD002 into *S. lividans* S4102 by conjugation, blue exconjugants appeared on MS plates containing erythromycin and nalidixic acid, indicating single-crossover mutants. The single-crossover mutants were inoculated on MS plates without antibiotics for sporulation. The generated spores were diluted and plated on MS plates containing apramycin. White colonies exhibiting an erythromycin-sensitive phenotype were verified as double-crossover mutants by PCR using the primer pair P07/P10 (Fig. S4). The double-crossover mutant with the markerless deletion of *int* was designated *S. lividans* S4104.

#### Construction of *S. lividans* S4106

The *S. lividans* strain S4106 was constructed by transforming pSET153 into *S. lividans* S4100 using the two-plasmid co-conjugation assay described above. PCR analysis was performed to confirm the integration of pSET153 in the resulting strains, using primer pairs P03/P07 and P08/P09, as shown in Fig. S7. The verified strain was designated *S. lividans* S4106.

#### Construction of S4108

The *S. lividans* strain S4108 was obtained by transforming pSET152::*idgS-sfp* into *S. lividans* S4100 through normal intergeneric conjugation. PCR analysis was then used to confirm the integration of pSET152::*idgS-sfp* in the resulting strains with primer pairs P03/P07 and P08/P09, as shown in Fig. S8. The verified strain was designated as *S. lividans* S4108.

#### Construction of S. lividans S4110

The *S. lividans* strain S4110 was constructed by transforming pSET153::*idgS-sfp* into *S. lividans* S4100 using the two-plasmid co-conjugation assay described above. PCR analysis was performed to confirm the integration of pSET153::*idgS-sfp* in the resulting strains, using primer pairs P03/P07 and P08/P09, as shown in Fig. S8. The verified strain was designated *S. lividans* S4110.

### Reverse transcriptase PCR (RT-PCR)

RNA samples were isolated from *S. lividans* S4102 and *S. lividans* S4104 grown in YEME medium at 28°C for 48 hours, using TRIzol reagent (Invitrogen). The absence of DNA contamination in the RNA samples was confirmed by PCR with the primer pair intinF/intinR. Complementary DNA (cDNA) was synthesized from 300 ng of total RNA using the PrimeScript RT Reagent Kit (TaKaRa Biotechnology Co., Ltd.) according to the manufacturer’s protocol. PCR was then performed using the generated cDNA as a template with the primer pair intinF/intinR. The products were detected by 1.0% agarose gel electrophoresis and visualized by staining with ethidium bromide.

### *In Vitro* recombination experiment

#### Protein expression and purification of recombinant Int

The recombinant plasmid pET22b::*int* was introduced into *E. coli* BL21(DE3) pLysS for protein expression. The Int protein was expressed as a C-terminal His_6_-tagged fusion protein. The Int protein was purified using a ProBond (Invitrogen) resin column according to the manufacturer’s protocol. Desalting and protein concentration were performed using an Amicon Ultra-15 Centrifugal Filter Unit (Millipore).

#### DNA substrate preparation

An *attP* fragment (801 bp) was generated by PCR using pSET152 as a template with the primer pair attPF/attPR. An *attB* fragment (1,214 bp) was amplified from the genomic DNA of *S. lividans* TK24 with the primer pair attBF/attBR. Both attP and attB fragments were used as substrates for the *in vitro attB* × *attP* recombination. pHyg::LR was prepared and used as the substrate for the *in vitro attL* × *attR* recombination.

#### *In Vitro* recombination assay

For *attB* × *attP* recombination, approximately 1 µg of each substrate DNA was mixed with 1 µg of purified Int in a 100 µL solution containing 20 mM Tris-HCl (pH 7.5), 100 mM NaCl, 1% glycerol, and 0.1 mM EDTA as described previously (17). The mixture was incubated at 30°C for 30 minutes. The reactions were terminated by phenol extraction and ethanol precipitation, and the pellets were resuspended in 50 µL of 1× reaction buffer. The concentrated products were separated by electrophoresis on 0.8% agarose gels to detect the recombination. *attL* × *attR* recombination *in vitro* was assayed with plasmid pHyg::LR as the substrate under the same conditions described above. Recombinant molecules were directly detected by PCR using the primer pair P07/P08, followed by agarose gel electrophoresis. The recombination was also detected by introducing the whole reaction mixture into *E. coli* JM109 *via* transformation and selecting transformants on media containing hygromycin.

### Determination of the frequency of excision in *Streptomyces*

The frequency of excision for the verified integrants derived from *S. lividans* S4100 was studied in the absence of antibiotic pressure. The spores of the verified integrant cells were plated on MS plates without antibiotics. After growing at 30°C for 5 days, the generated spores were pooled by washing them off the plates with 1 mL of 1× phosphate-buffered saline (PBS) and subsequently diluted in PBS. Appropriate dilutions were plated on MS plates either without antibiotics (to determine the total number of cells) or with hygromycin (to determine the number of cells that had lost the integration vector and thus become resistant to hygromycin). The frequency of excision was estimated as follows: (the number of cells that had lost the integration vector / the total number of cells) × 100%. Each experiment was repeated independently at least three times. To investigate whether excision took place, genomic DNA was prepared from the relevant hygromycin-resistant strains and used as the template in PCRs. The primers used were P07/P08, which span the *attB* region on *hygB* (Table S2).

For the indigoidine-producing strain created in this study, the frequency of excision during fermentation was also monitored using a similar strategy. The mycelium pellets in the culture at each time point were collected and plated on MS plates for sporulation without the addition of antibiotics. After sporulation, appropriate dilutions of spores were plated on MS plates either without antibiotics (to determine the total number of cells) or with hygromycin (to determine the number of cells that had become resistant to hygromycin). The frequency of excision was estimated as follows: (the number of cells that had lost the integration vector / the total number of cells) × 100%.

### Measurement of indigoidine production

Spore suspensions were inoculated in 10 mL of TSB liquid media with apramycin and incubated at 28°C on a rotary shaker (220 rpm) for 48 hours as a seed culture. About 1 mL of the seed culture was then transferred into 50 mL of TSB liquid media supplemented with 10 g/L glucose and cultured in a shaking incubator (28°C, 200 rpm) without antibiotics. After incubation for 24, 48, 72, 96, or 120 hours, the indigoidine titer was measured using a colorimetric assay as previously described (45). Briefly, 100 µL of the fermentation broth was pelleted by centrifugation at 20,000 × *g* for 2 minutes. The supernatant was discarded, and 500 µL of DMSO was added to the pellet. The solution was sonicated and vortexed vigorously for 10 minutes to dissolve the indigoidine. After centrifugation at 20,000 × *g* for 2 minutes, 100 µL of the dimethyl sulfoxide (DMSO)-extracted indigoidine was added to 96-well flat-bottomed microplates. Indigoidine was quantified by measuring the optical density (OD_612_) using an Infinite 200 PRO microplate reader and applying a standard curve generated from indigoidine standard with known concentrations.

### Statistical analyses

All experiments were conducted in triplicate. Mean values were compared using a two-tailed, unpaired Student’s *t-*test. *P* values of less than 0.05 were considered statistically significant.

## SUPPLEMENTAL MATERIAL

Supplemental material aem.01812-24-s0001.docx Tables S1 and S2; Figures S1 to S8.

ASM does not own the copyrights to Supplemental Material that may be linked to, or accessed through, an article. The authors have granted ASM a non-exclusive, world-wide license to publish the Supplemental Material files. Please contact the corresponding author directly for reuse.
