# Supplementary material for: Mitigating genetic instability caused by the excision activity of the phiC31 integrase in Streptomyces
Source: Appl Environ Microbiol. 2024 Dec 20;91(1):e01812-24. doi: 10.1128/aem.01812-24 (PMC11784100; doi:10.1128/aem.01812-24)
Supplement: Supplemental material — Tables S1 and S2; Figures S1 to S8. [file aem.01812-24-s0001.docx]

**Supplementary materials**

Table S1. Plasmids and strains used in this study.

| Plasmids /strains | Genotype/phenotype^a^ | Source |
| --- | --- | --- |
| plasmids |  |  |
| pUC57 | *E. coli* cloning vector, Kan^r^ | Genewiz |
| pUC57::*hyg* | pUC57 containing hygromycin resistance gene,Kan^r^, Hyg^r^ | This study |
| pHygB-123 | pUC57::*hyg* derivative with the *attB* insertion after codon 123^rd^ of *hyg.*  Kan^r^ | This study |
| pHygB-135 | pUC57::*hyg* derivative with the *attB* insertion after codon 135^th^ of *hyg.* Kan^r^, Hyg^r^ | This study |
| pHygB-168 | pUC57::*hyg* derivative with the *attB* insertion after codon 168^th^ of *hyg*, Kan^r^ | This study |
| pHygB-215 | pUC57::*hyg* derivative with the *attB* insertion after the codon 215^th^ of *hyg*, Kan^r^, Hyg^r^ | This study |
| pHyg::LR | pUC57::*hyg* derivative with the insertion of a fragment flanking with *attL* and *attR* into *hyg* after codon 135^th^ , Kan^r^ | This study |
| pET22b | His_6_ fusion protein expression vector, Amp^r^ | This study |
| pET22b:*:int* | Int expression plasmid, Amp^r^ | This study |
| pSET152 | *Streptomyces* integration vector, Apr^r^ |  |
| pSET153 | pSET152 derivative with the deletion of *int*, Apr^r^ | This study |
| pINT01 | pUC19::*kasO*p*::*int*::*oriT*, Amp^r^ | This study |
| pSET152::*idgs*-*sfp* | pSET152:::*ermE*p*::*idgs-sfp*, Apr^r^ | This study |
| pSET153::*idgs*-*sfp* | pSET153:::*ermE*p*::*idgs-sfp*, Apr^r^ | This study |
|  |  |  |
| pCIMt004 | A *Streptomyces* suicide vector containing *idgS*-*sfp* operon, Amp^r^, Erm^r^ | (1) |
| pDYD001 | pCIMt004 directives used for *attB* replacement with *hygB*, Amp^r^, Erm^r^,Hyg^r^ | This study |
| pDYD002 | pCIMt004 directives used for *int* deletion, Amp^r^, Erm^r^ | This study |
| Strains |  |  |
| *S. lividans* TK24 | *S. lividans* wild type | (2) |
| *S. lividans* S4098 | pSET152/TK24, Apr^r^ | This study |
| *S. lividans* S4100 | Δ*attB*, TK24::*hygB,* Hyg^r^ | This study |
| *S. lividans* S4102 | pSET152/S4100, Apr^r^ | This study |
| *S. lividans* S4104 | Δ*int*/S4102, Apr^r^ | This study |
| *S. lividans* S4106 | pSET153/ S4100, Apr^r^ | This study |
| *S. lividans* S4108 | pSET152::*idgs*-*sfp*/S4100, Apr^r^ | This study |
| *S. lividans* S4110 | pSET153::*idgs*-*sfp*/S4100, Apr^r^ | This study |

^a^Note*:* Kan^r^: Kanamycin resistance; Hyg^r^: Hygromycin B resistance; Amp^r^: Ampicillin resistance; Apr^r^: Apramycin resistance; Erm^r^ : Erythromycin resistance

Table S2. Primers used in this study.

| Primers | | Sequence 5’->3’ | | | Purpose |
| --- | --- | --- | --- | --- | --- |
| pUC57F-BsaI | | | AGGGTCTCATCGCGAATGGCGCCTGATGCGG | pUC57::*hyg construction* | |
| pUC57R-BsaI | | | ACGGTCTCTACTGGTCATAGCTGTTTCCTGT | pUC57::*hyg construction* | |
| hygF | | | GGGTCTCACAGTCCCCGACGTGGCCGACCA | pUC57::*hyg construction* | |
| hygRDBsaI | | | AGGGTCTCAGGTTTCCTCGAACACCTCGAAGTC | pUC57::*hyg construction* | |
| hygFDBsaI | | | ACGGTCTCTAACCCCGCTGGATCTCTCCG | pUC57::*hyg construction* | |
| hygR | | | ACGGTCTCTGTGACGGGGCTCTTCACCCGGG | pUC57::*hyg construction* | |
| 123F-attB | | | AGGGTCTCACCTTGGGCTCCCCGGGCGCG GGTCATCCGGCTCATCACCA | pHygB-123 construction | |
| 123R-attB | | | ACGGTCTCTAAGGGCACGCCCTGGCACCC GGCACCACCTGGCGGTCCGC | pHygB-123 construction | |
| 135F-attB | | | AGGGTCTCACCTTGGGCTCCCCGGGCGCG GGTCGTGCCGTCCATCGCGG | pHygB-135 construction | |
| 135R-attB | | | ACGGTCTCTAAGGGCACGCCCTGGCACCC GACCGGAACGCGCTGCTCGC | pHygB-135 construction | |
| 168F-attB | | | AGGGTCTCACCTTGGGCTCCCCGGGCGCG ATGGGGGGTGAGCACGGTGT | pHygB-168 construction | |
| 168R-attB | | | ACGGTCTCTAAGGGCACGCCCTGGCACCC TCCGAGGTCTTCCCGGAACT | pHygB-168 construction | |
| 215F-attB | | | AGGGTCTCACCTTGGGCTCCCCGGGCGCG CGGCCGGCCAGCAGCGTGT | pHygB-215 construction | |
| 215R-attB | | | ACGGTCTCTAAGGGCACGCCCTGGCACCC GAACCCCGGTTCGTCCACGG | pHygB-215 construction | |
| attBHR | | | ACGGTCTCTAAGGGCACGCCCTGGCACCCGAAC | pHyg::LR construction | |
| attBHF | | | AGGGTCTCATTGGGCTCCCCGGGCGCGGCGGC | pHyg::LR construction | |
| attPHF | | | AGGGTCTCACCTTGAGTTCTCTCAGTTGGGGGCGTA | pHyg::LR construction | |
| inR-HindIII | | | AGGGTCTCAGCTTTGGGCTTGCTGCGCTCGA | pHyg::LR construction | |
| inF-hindIII | | | ACGGTCTCTAAGCTTAAGCTCTAGCGATTCCAGACG | pHyg::LR construction | |
| attPHR | | | ACGGTCTCTCCAAAGGTTACCCCAGTTGGGGCAC | pHyg::LR construction | |
| intF-NdeI | | | TATACATATGACACAAGGGGTTGTGACC | pET22b::*int* construction | |
| intR-xhoI | | | TGCTCGAGCGCCGCTACGTCTTCCGT | pET22b::*int* construction | |
| oriTF | | | AGGAATTCTCATGGCTCTGCCCTCGG | pINT01 construction | |
| oriTR | | | AGGGTACCCCAAAGGGTTCGTGTAGAC | pINT01 construction | |
| kasOF | | | TCGGTACCCGGTGTTCACATTCGAACGGT | pINT01 construction | |
| kasOR | | | ACTCTAGAAACTCCCCCAGTCCTGCA | pINT01 construction | |
| intF-XbaI | | | AGTCTAGAATGACACAAGGGGTTGTGACC | pINT01 construction | |
| intR-SphI | | | AGGCATGCGGTGTCTCGCTACGCCGCTA | pINT01 construction | |
| ermF | | | CAGGTCGACTCTAGAGGATCAGCCCGACCCGAGCACGCGC | pSET152::*idgS*-*sfp,*  pSET153::*idgS*-*sfp* construction | |
| sfpR | | | GCCGGGTTCCCGGGTCTAGACAAGCTTTTATAAAAGCTCT | pSET152::*idgS*-*sfp,*  pSET153::*idgS*-*sfp* construction | |
| tfd-F | | | AGAGCTTTTATAAAAGCTTGTCTAGACCCGGGAACCCGGC | pSET152::*idgS*-*sfp,* pSET153::*idgS*-*sfp* construction | |
| tfd-R | | | ATCGCGCGCGGCCGCGGATCAAAGTTTTGTCGTCTTTCCAGACG | pSET152::*idgS*-*sfp,*  pSET153::*idgS*-s*fp* construction | |
| attBupF | | | AAAGCTTCCATGGGCACGCCGCGAGGACGTAGTGGAGGATG | pDYD001 construction | |
| attBupR | | | TGGTCGGCCACGTCGGGGACACTCCACCTCACCCATCTGG | pDYD001 construction | |
| hygF-up | | | CCAGATGGGTGAGGTGGAGTGTCCCCGACGTGGCCGACCA | pDYD001 construction | |
| hygR-dn | | | GTCTCGAAGCCGCGGTGCGGACGGGGCTCTTCACCCGGG | pDYD001 construction | |
| attBdnF | | | CCCGGGTGAAGAGCCCCGTCCGCACCGCGGCTTCGAGAC | pDYD001 construction | |
| attBdnR | | | TAGCATCGGGCGCAACCTAGGTACGGCTCTGGATCATCGG | pDYD001 construction | |
| intupF | | | AAAGCTTCCATGGGCACGCCGTGCCGAGCAGCGCGAGCAG | pDYD002 construction | |
| intUpR | | | CGCTACGTCTTCCGTGCCGTCTTGTCTTCGTTGGCGCTAC | pDYD002 construction | |
| intdnF | | | GTAGCGCCAACGAAGACAAGACGGCACGGAAGACGTAGCG | pDYD002 construction | |
| intdnR | | | TAGCATCGGGCGCAACCTAGCGTGAGTTTTCGTTCCACTGAG | pDYD002  construction | |
| attPF | | | GTGATCTCCTTCGTCTCCGA | *attP* fragment preparation | |
| attPR | | | CGAAGGCGTGGCGCGGCTTC | *attP* fragment preparation | |
| attBF | | | GCTCCGGGGCCCTCGCCCGA | *attB* fragment preparation | |
| attBR | | | GACCTGCTGCCGACCGGCTA | *attB* fragment preparation | |
| intinF | TTGGCGCTTCTGTGGGAAGC | | | RT-PCR | |
| intinR | GCCGGTCGGGTCAGCGTCGG | | | RT-PCR | |
| P01 | CGCCAAAGCTGTCGAACA | | | S4098 verification | |
| P02 | ATGGCCCGTACTGACGGAC | | | S4098 verification | |
| P03 | CCCCCGTCGCGCTCGACTTCGC | | | S4098, S4102, S4106, S4108, S4110 verification | |
| P04 | AGGCCGTTGAAGTCCTCCCG | | | S4098 verification | |
| P05 | TGTGCTCGACGTTGGTGGC | | | S4100 verification | |
| P06 | GCGAGGGCAAGAGCATCTGGTA | | | S4100 verification | |
| P07 | AAGGTGAAGGCGAGCAGTT | | | L X R Recombination detection; S4102, S4104, S4106, S4108, S4110 verification | |
| P08 | ACCCGGTGATCAAGCTGTT | | | L X R Recombination detection; S4102, S4106, S4108, S4110 verification | |
| P09 | CAATCGCTCTTCGTTCGTCT | | | S4102, S4106, S4108, S4110 verification | |
| P10 | AGCAACGCGGCCTTTTTACG | | | S4104 verification | |

**Figure S1**. Genetic heterogeneity of the pSET152 integration strain *S. lividans* S4098 during cultivation without antibiotic pressure. (A) Illustration of the integration of pSET152 into the *attB* site of the *S. lividans* TK24 genome to generate strain S4098. (B) PCR verification of pSET152 integration in strain S4098. (C) Amplification of a DNA fragment corresponding to the wild-type *attB* locus from the genomic DNA of S4098 grown without antibiotic pressure, indicating genetic heterogeneity in *S. lividans* S4098 during cultivation


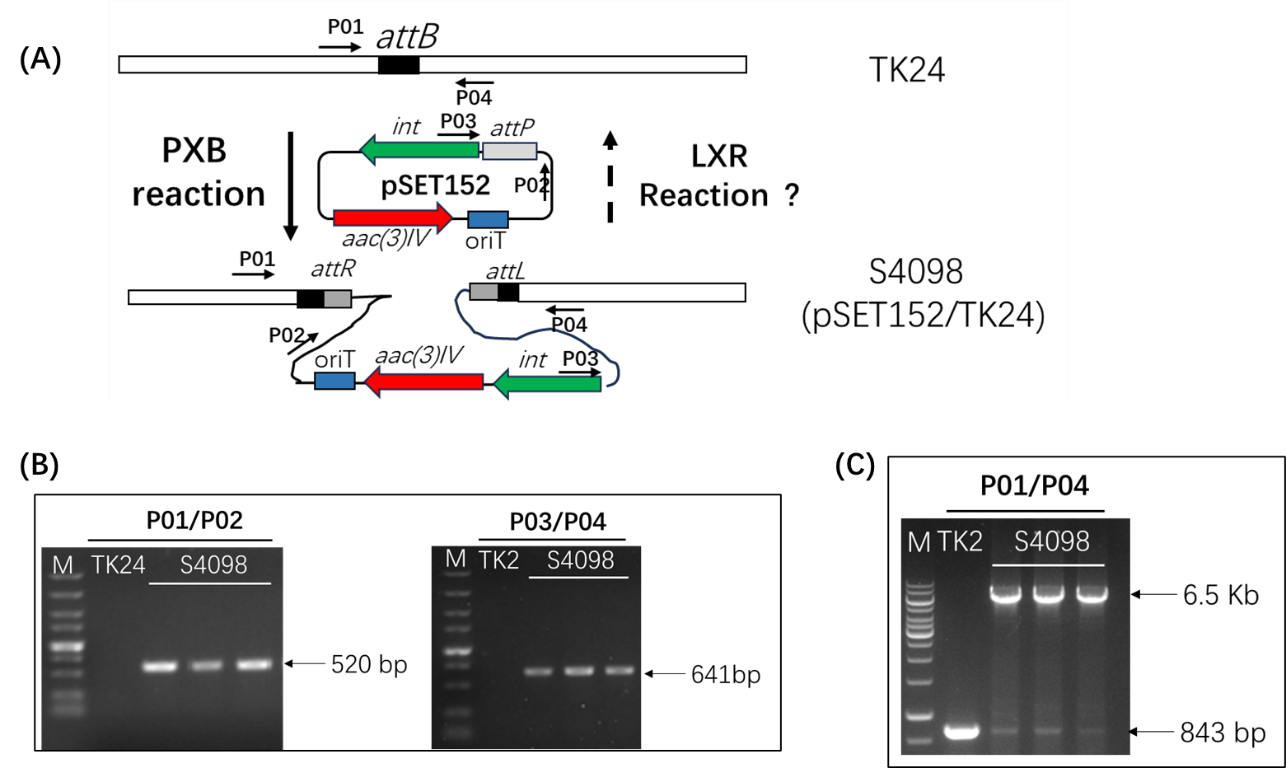


**Figure S2.** Construction of strain S4100. (A) Schematic diagrams of homologous recombination. (B) Results of PCR verification for the construction of S4100.


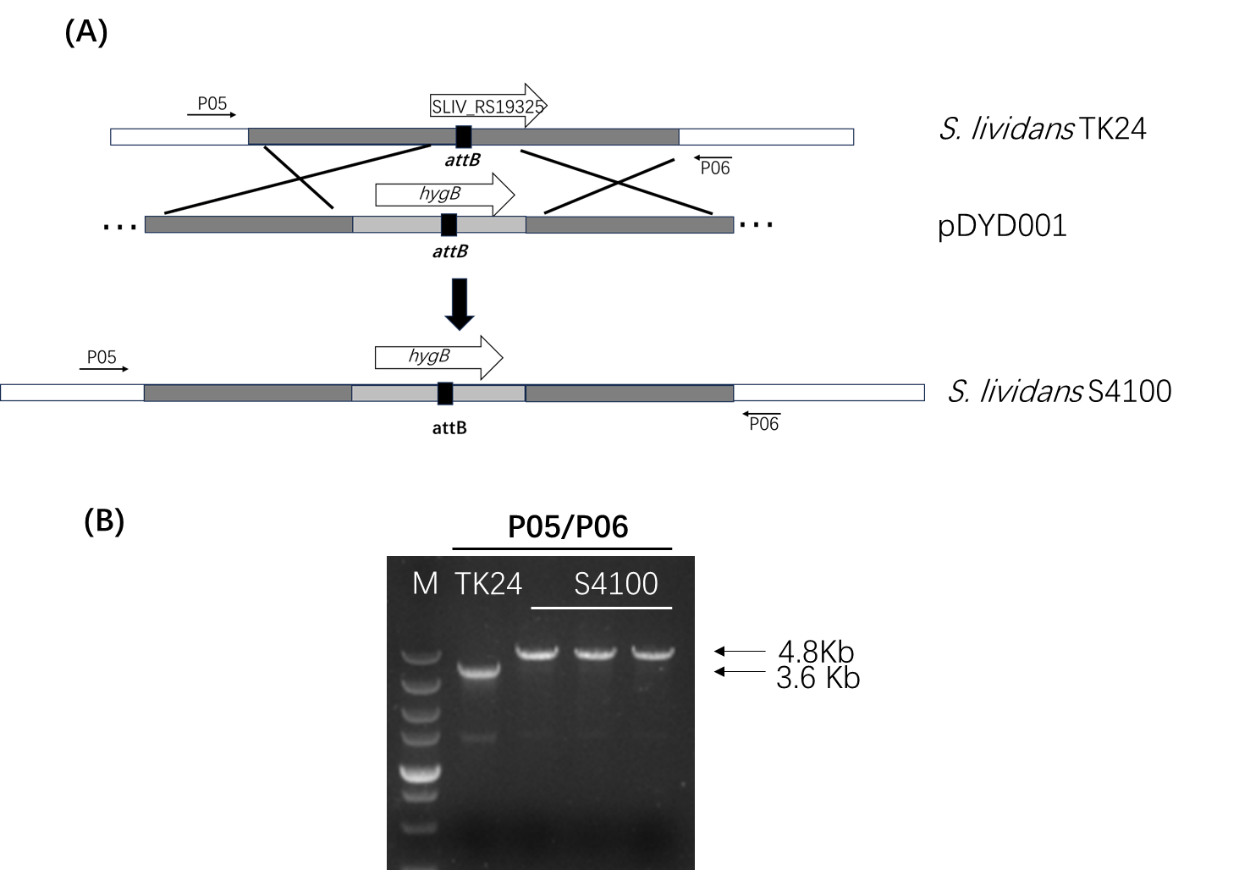


**Figure S****3.** Construction of strain S4102. (A) Illustration of the integration of pSET152 into the *attB* site of the genome of *S. lividans* S4100 to obtain *S. lividans* S4102. (B) PCR verification of pSET152 integration in *S. lividans* S4102.


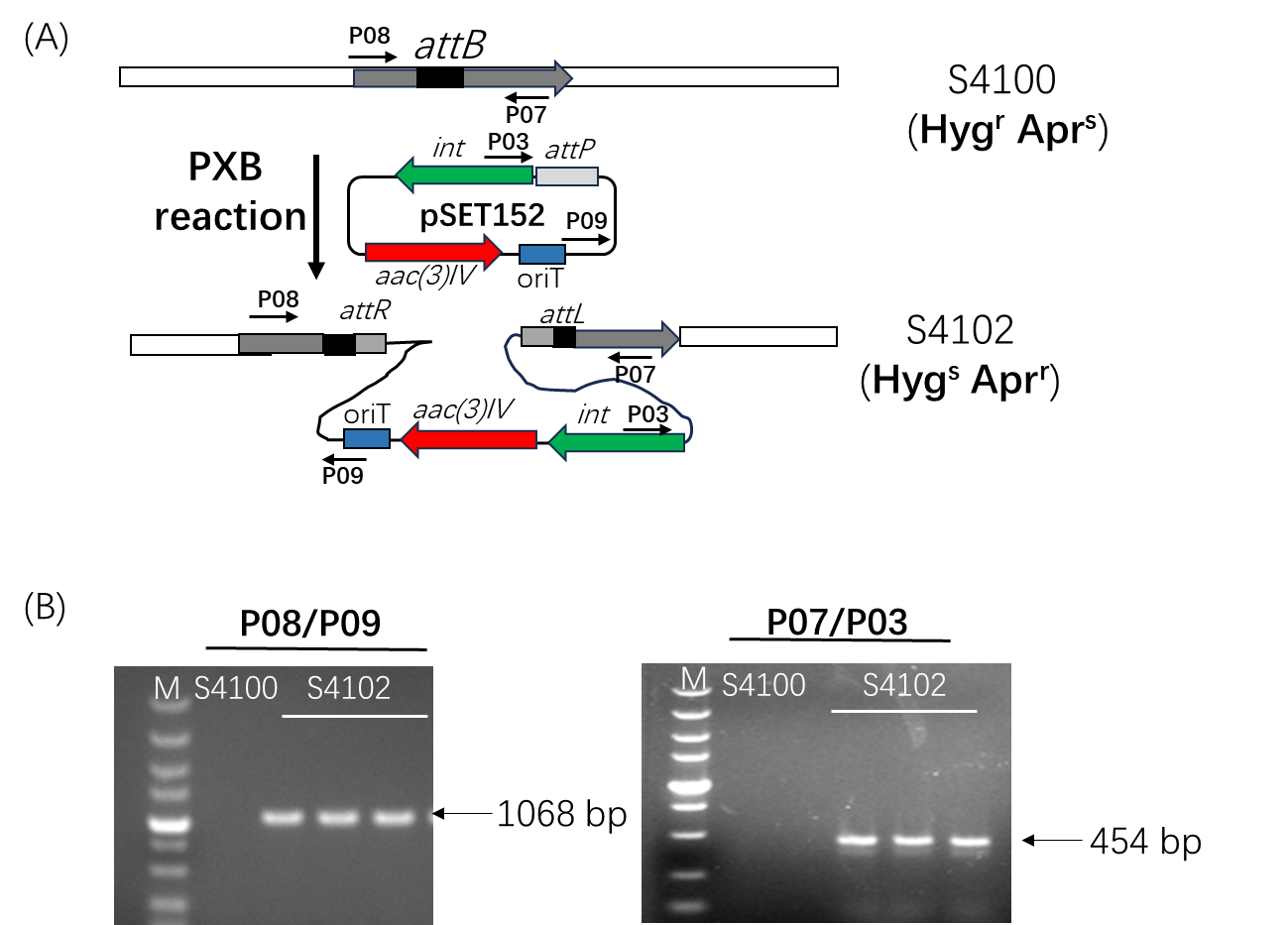


**Figure S4**. Construction of *int* deletion strain *S. lividans* S4104. (A) Illustration of the deletion of *int* *from* S4102 genome. (B) PCR verification of *int* deletion in *S. lividans* S4104 .


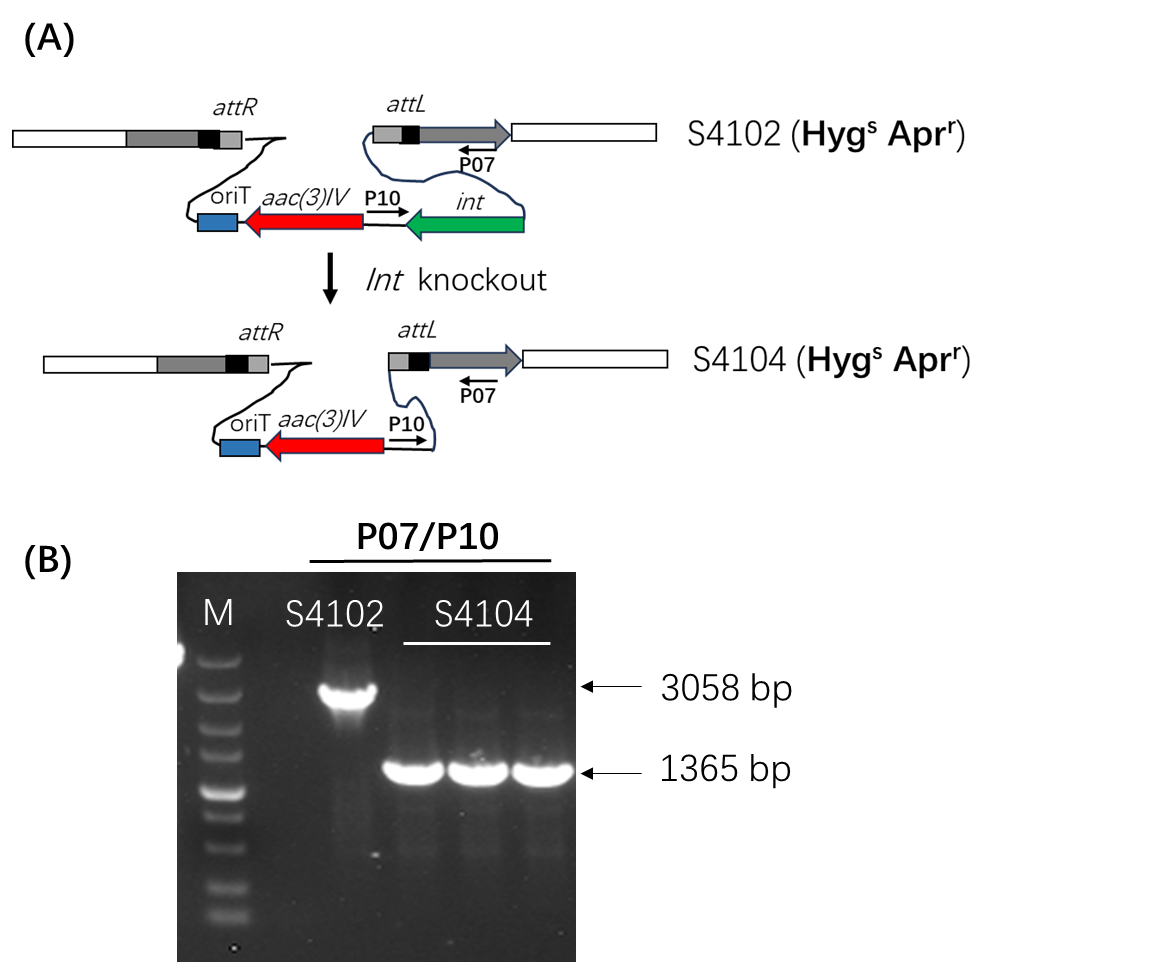


**Figure S5**. Transcriptional analysis of *int* by RT-PCR. M : Marker, *int* specific primers (intinF/intinR) were used for the PCR from S4102 cDNA ( lane1, 2,3), and S4104 cDNA (lane 4, 5,6).


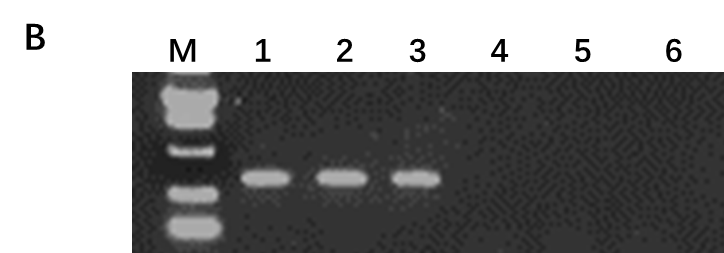


**Figure S6.** *In vitro* recombination assay using purified Int. (A) SDS-PAGE of recombinant *phi*C31 Int. Lane M: protein marker; Lane 1: C-His_6_-tagged Int. (B) Recombination by *phi*C31 Int between *attP* and *attB* sites *in vitro*. Parental substrates and expected recombinant products are indicated in the left panel. Standard recombination conditions were applied, and recombination products were detected directly by agarose gel electrophoresis. (C) Recombination by *phi*C31 Int between *attL* and *attR* sites *in vitro*. Parental substrates and expected recombinant products are indicated in the left panel. Recombinant molecules were detected by PCR with primer pair P07/P08 and agarose gel electrophoresis (right panel). No recombinant molecules were detected.


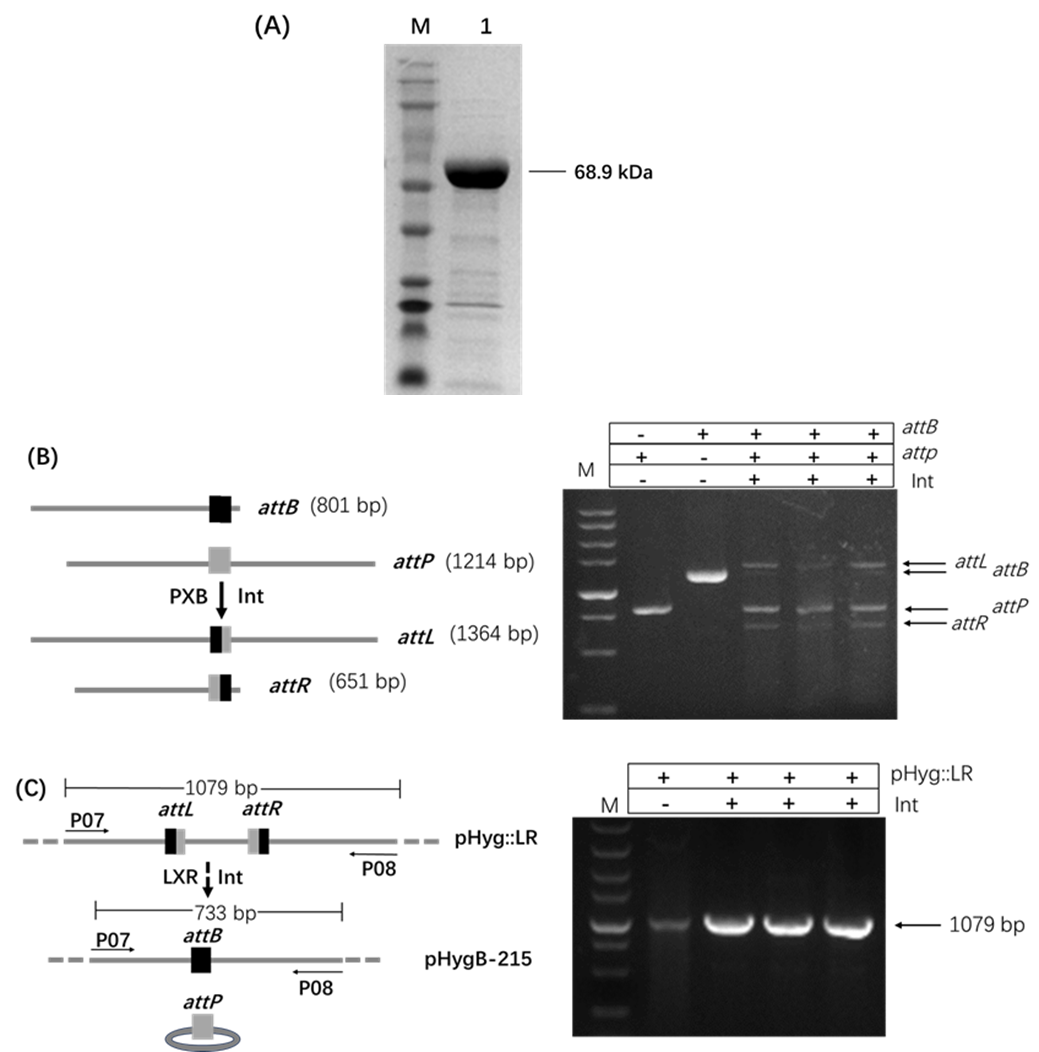


**Figure S7.** Construction of *S. lividans* S4106. (A) Illustration of the integration of pSET153 into the *attB* site of the genome of *S. lividans* S4100 to obtain *S. lividans* S4106; (B) PCR verification of pSET153 integration in Strain S406*.*


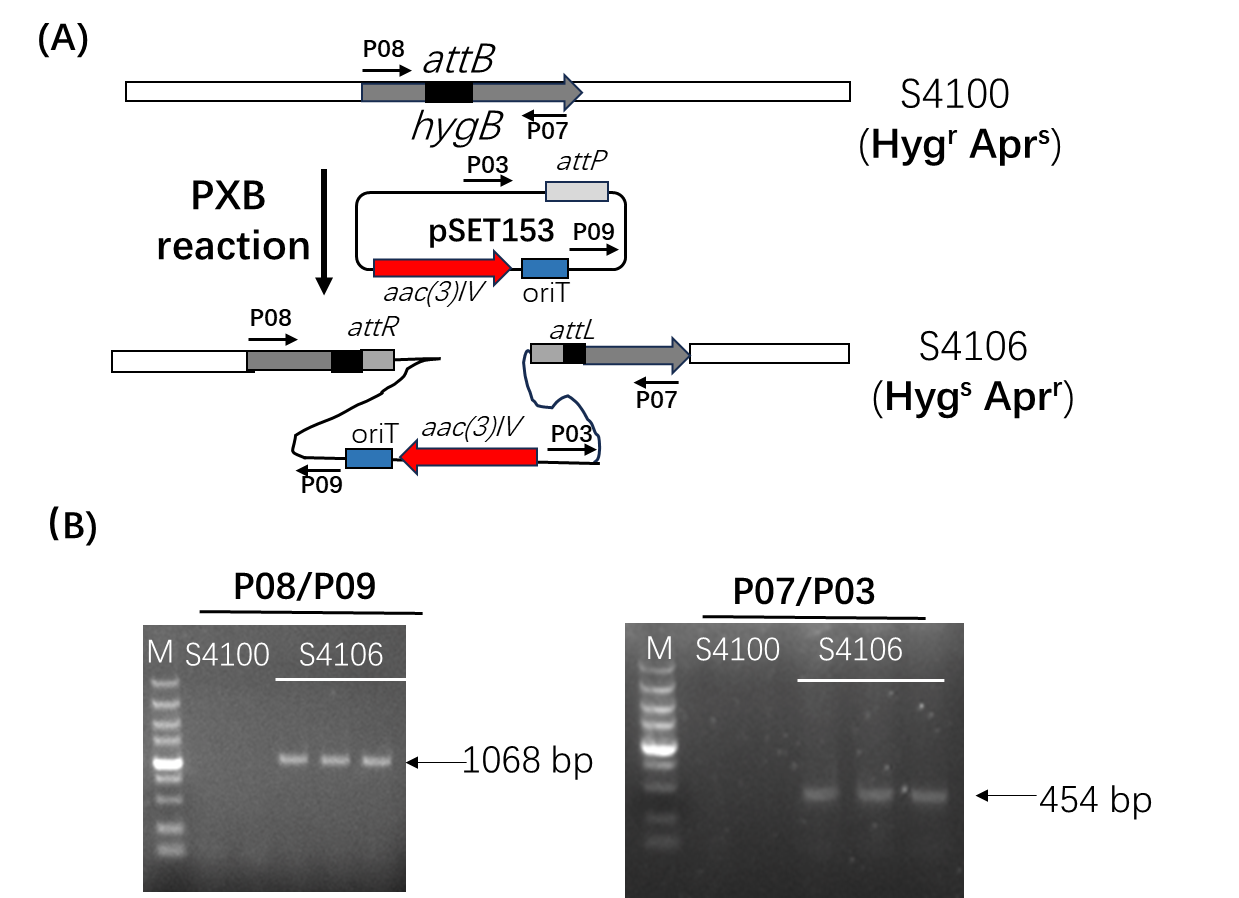


**Figure S8.** Construction of strain *S. lividans* S4108 and *S. lividans* S4110. (A) Illustration of the integration of pSET152::*idgS-sfp* into the *attB* site of the genome of *S. lividans* S4100 to obtain *S. lividans* S4108 .(B) Illustration of the integration of pSET153::*idgS-sfp* into the *attB* site of the genome of *S. lividans* S4100 to obtain *S. lividans* S4110. (C) PCR verification of plasmid integration in Strain S4108 and S4110.


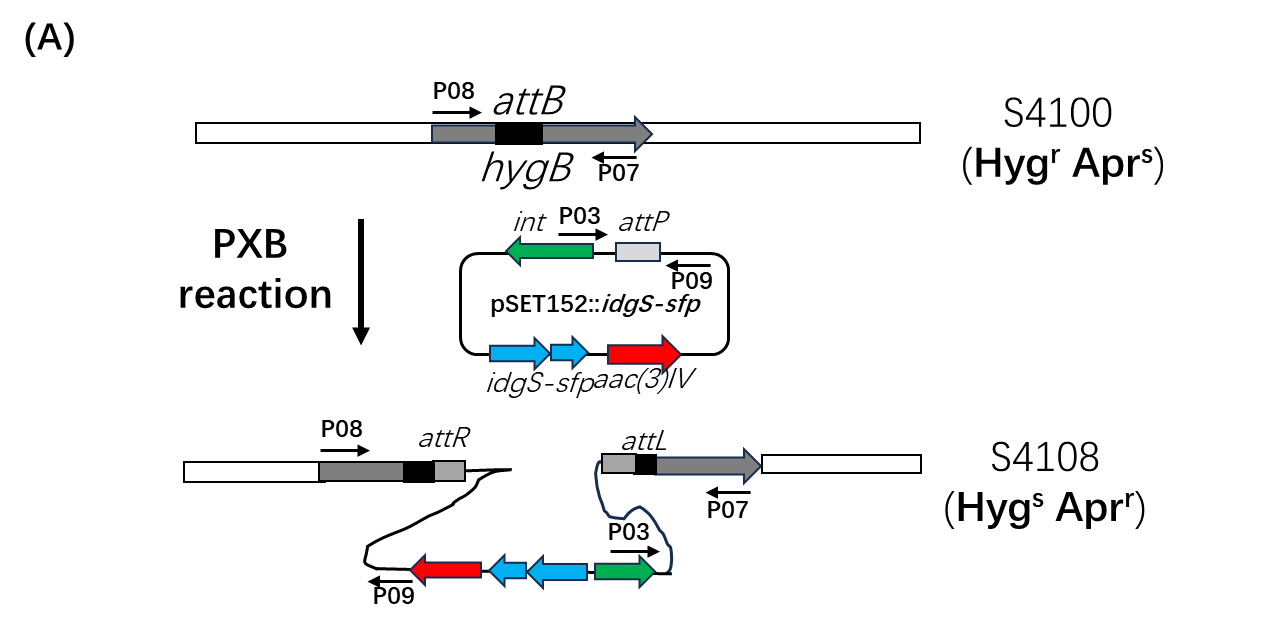


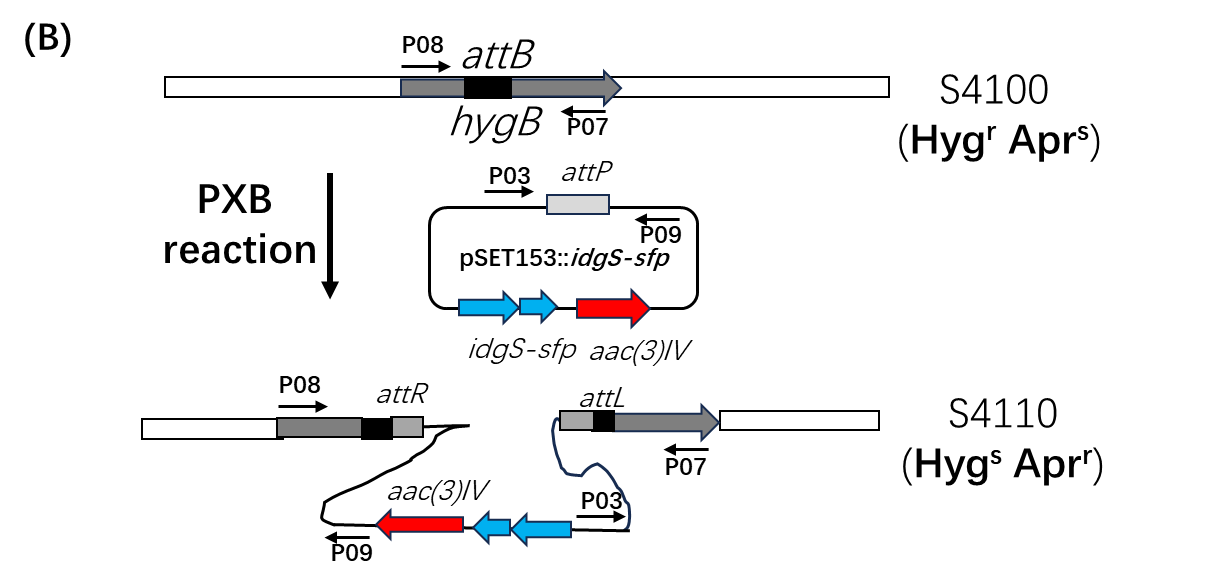


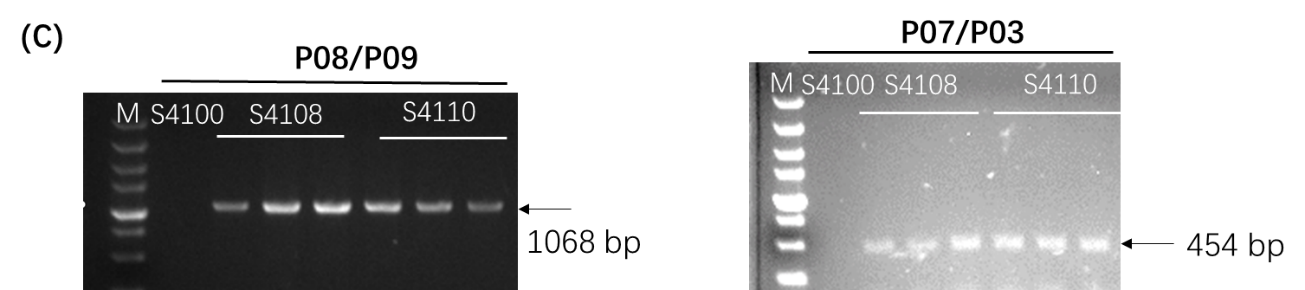


**References**

1. Li P, Li J, Guo Z, Tang W, Han J, Meng X, Hao T, Zhu Y, Zhang L, Chen Y. 2015. An efficient blue-white screening based gene inactivation system for Streptomyces. Appl Microbiol Biotechnol 99:1923-33.

2. Ruckert C, Albersmeier A, Busche T, Jaenicke S, Winkler A, Friethjonsson OH, Hreggviethsson GO, Lambert C, Badcock D, Bernaerts K, Anne J, Economou A, Kalinowski J. 2015. Complete genome sequence of Streptomyces lividans TK24. J Biotechnol 199:21-2.
